# Barriers and Facilitators of Romanian HPV (Human Papillomavirus) Vaccination

**DOI:** 10.3390/vaccines10101722

**Published:** 2022-10-15

**Authors:** Loredana Sabina Cornelia Manolescu, Corina Zugravu, Corneliu Nicolae Zaharia, Anca Irina Dumitrescu, Irina Prasacu, Mihaela Corina Radu, Goraneanu Draghici Letiția, Irina Nita, Corina Marilena Cristache, Laurentia Nicoleta Gales

**Affiliations:** 1 Department of Microbiology, Parasitology and Virology, Faculty of Midwifery and Nursing, “Carol Davila” University of Medicine and Pharmacy, 050474 Bucharest, Romania; 2 Department of Virology, Institute of Virology “Stefan S. Nicolau”, 030304 Bucharest, Romania; 3 Department of Fundamental Sciences, Faculty of Midwifery and Nursing, University of Medicine and Pharmacy, Carol Davila, 37 Dionisie Lupu St., 020021 Bucharest, Romania; 4 National Center for Monitoring the Community Risks, Institute of Public Health, 1-3 Doctor Leonte Anastasievici St., 050463 Bucharest, Romania; 5 Department of Fundamental Sciences, Faculty of Pharmacy, “Carol Davila” University of Medicine and Pharmacy, 050474 Bucharest, Romania; 6 Department of Birth, Obstetrics and Gynecology Hospital, 100409 Ploiesti, Romania; 7 Medical Oncology Department, Monza Oncology Hospital, 011461 Bucharest, Romania; 8 Department of Dental Techniques, Faculty of Midwifery and Nursing (FMAM), “Carol Davila” University of Medicine and Pharmacy, 8 Eroilor Sanitari Blvd., 050474 Bucharest, Romania; 9 Department of Oncology, Institute of Oncology “Prof. Dr. Al. Trestioreanu” Bucharest, Faculty of Medicine, “Carol Davila” University of Medicine and Pharmacy, 050474 Bucharest, Romania

**Keywords:** human papillomavirus, HPV vaccination, children vaccination

## Abstract

Background: Many countries had initial success with HPV vaccination campaigns worldwide. The HPV vaccine coverage during the COVID-19 pandemic dropped consistently. The aim of our research is to assess the barriers and facilitators of the current Romanian HPV vaccination campaign. Methods: An analytical cross-sectional observational survey was conducted in the Romanian general population; a self-administered questionnaire was used. Results: 1122 responders were interviewed; 666 (59.36%) were parents, and 67 (5.97%) HPV vaccinated themselves. A multinominal logistic regression carried out in the parents’ category showed that women with university studies and informed from medical sources have greater chances to HPV vaccinate. Reticence regarding vaccination comes from the high cost of the vaccine and a lack of information. Only 118 (10.51%) vaccinated against HPV. From the logistic regression analysis, gender (ORa 0.461 = 95% CI: (0.196; 1.083)), geographic area of residence (ORa = 0.517; 95% CI: (0.331; 0.807)), and the inclusion of the HPV vaccine in the National Vaccination Program (ORa = 2.4; 95% CI: (1.361; 4.235)) were the factors found most associated with HPV vaccination. Conclusions: In the general population, the inclusion of HPV vaccination in the National Vaccination Program would be the most important facilitator of HPV vaccination in Romania. In parents that did not vaccinate their children, one important barrier to HPV vaccination was the high cost of the HPV vaccine and the lack of proper information. The acceptability of HPV vaccination in Romania is low; therefore, public health educational programs are needed.

## 1. Introduction

Human papillomavirus (HPV) infection is well-known as being the number one sexually transmitted viral infection in the world. Almost every sexually active person comes into contact with at least one of the strains of the HPV virus in the course of their life [1]. HPV causes cervical, head and neck, and anogenital cancers, with huge lifetime medical costs of chronic conditions worldwide [2]. Even though approximately 5% of the world’s cancers are specifically attributed to HPV, gender-neutral HPV vaccination is lacking or delayed in many countries [3]. A gender-neutral HPV vaccine implementation strategy is the most effective way—even at moderate coverage—to meet the World Health Organization’s goal of eradicating cervical cancer [4,5]. Furthermore, herd immunization effects [6] are beneficial for preventing infections in unvaccinated adolescents and young adults; these are improved policies and methods to build awareness and enhance the acceptance of vaccine programs in all countries. The prevalence of HPV-associated cancers differs and is influenced by the HPV vaccination rate. Access to HPV vaccination and regular screening for HPV-associated cancer prevents people from becoming infected with HPV, the primary cause of cervical cancer worldwide [3,7,8]. Over time, the wide availability of HPV vaccination and screening in high-income countries has significantly contributed to the low mortality rate from HPV-related cancers compared with low-income countries [9]. Although many countries had successful HPV vaccination campaigns since 2006 when the first HPV vaccine was released [10], worldwide HPV vaccine coverage during the COVID-19 pandemic has dropped consistently [11].

The Romanian HPV vaccination experience started in 2008, and until 2020, it was never a success story due mainly to the low level of information [1,12]. The Romanian Ministry of Health started, in 2020, a new HPV vaccination campaign, constantly upgrading and adapting to the needs of the Romanian population [13], as according to current data, cervical cancer is the second most-encountered cancer in Romanian women aged 15 to 44 years [14]. In the present in Romania, routine HPV vaccination is recommended from age 11, (it can be started at age 9), to age 26 (for adults aged 27 through 45 years, HPV vaccination can be considered if beneficial). [10]. In Romania, HPV vaccination is possible only with parental consent until 18 years of age, due to the fact that HPV vaccination is not included in the National Vaccination Program [15]. As for the legal framework, family doctors are responsible for HPV vaccination in Romania, including access to free HPV vaccines, which are provided by the Ministry of Health at their request. Vaccination is recommended for girls and boys as well. As for the public attitudes and potential hesitant attitudes, several Romanian studies regarding vaccination have shown that a low level or absence of knowledge has determined parents’ decision to not vaccinate their children in general [16,17,18,19]. It is known and proven that HPV vaccination is an effective tool against several types of cancer, especially cervical cancer. In Romania, the HPV vaccination campaigns have failed; therefore, the aim of our research is to assess the barriers and facilitators of the present HPV vaccination Romanian campaign and the degree of acceptability, knowledge, and intention of Romanian parents to vaccinate their children in order that the present HPV vaccination campaign should be a success.

## 2. Materials and Methods

### 2.1. Study Design, Setting, and Participants

An analytical cross-sectional observational survey was conducted in Romania in the general population. For an accurate design, we used “The Strengthening the Reporting of Observational Studies in Epidemiology” (STROBE) recommendations in our observational study. The STROBE checklist is included in the Supplementary Materials (Supplementary Materials File S3).

The study was conducted with the aid of volunteers, mainly medical students or former graduates of the “Carol Davila” University of Medicine and Pharmacy from Bucharest in Romania in all 41 counties and in the capital, Bucharest. The volunteers explained the questionnaire to the general population to achieve accurate answers.

This study was addressed to the general Romanian population, all people living in the Romanian counties and the capital. The goal of attendance was at least 1000 participants and at least 25 answers from each county. The inclusion criteria for the participants were to be at least 18 years of age and to have Romanian citizenship. The participants were contacted by volunteers, and everybody was welcomed. The domain of activity of the participants was defined as employed, unemployed, student, or employed in the medical domain. The activity in the medical domain included medical doctors, nurses, pharmacists, paramedics, and midwives. We considered that people working in the medical domain may impact the general population’s vaccination acceptability. The level of education was reflected by the level of completed studies, including high school, post-secondary school, and university/post-university studies.

### 2.2. Survey Questionnaire and Data Collection

The data were collected with the use of a self-administered questionnaire, and informed consent was required and obtained from each willing participant. The questionnaire was administered online using the Google Forms cloud-based survey software and was available online for four months: March, April, May, and June in 2022. It included 14 multiple-choice questions spread into a general data section and an HPV vaccine information section (Supplementary Materials File S1). After it was issued in Google Forms, informed consent was required and obtained from each willing participant, and the responder was initially informed of the purpose of the survey. All questions were mandatory, the questionnaire could only be submitted after all its questions were answered.

### 2.3. Ethical Approval

The questionnaire was peer-validated and approved by the Ethics Committee of the Obstetrics and Gynecology Hospital, Ploiesti, Romania (41482/09.08.2022); all the procedures in the study respected the ethical standards of the Helsinki Declaration. Informed consent was compulsory.

### 2.4. Statistical Analysis

The data from the questionnaire were analyzed by means of the Microsoft Office package Excel and IBM^®^ SPSS^®^ Statistics Version 23.0 software. For data processing, the COUNTIFS function in Excel was used to filter and sort the initial database. The decision regarding vaccinating children (no intention, intention, already vaccinated) in people having children was considered the most important variable and was, hence, considered as the main dependent variable for consecutive analysis. Applied tests were descriptive ones. Since all data, except for age, were discrete variables, we applied also the non-parametric Kruskal–Wallis test. Age was tested in a correlation test (Spearman Rho, since age distribution is not normal). The variables considered significant in relation to our main dependent variable were included in a logistic regression to identify the most important predictors for deciding to vaccinate children. For all tests, the threshold for statistical significance was considered to be 0.05.

## 3. Results

In total, 1122 responders were interviewed. The answers to the questions are summarized in the Supplementary Materials File S2. The data gathered in the first part of the study revealed the following profile of the group: mean age 36.39 ±10.7 years (limits: 19–62), majority women, 999 (89.04%), and from urban areas, 930 (82.89%). Most of the participants in the survey (684 (60.96%)) had university and post-university studies, and 459 (40.91%) participants were related to the medical domain. The general characteristics of the participants in the survey are summarized in Table 1. It is very clear that a small percentage of our group is vaccinated against HPV: only 118 (10.51%) of the participants in the survey.

In the whole studied group, there were 666 (59.36%) parents; i.e., participants that had children of various ages. There were 39 (3.48%) parents that had children aged less than 9 years old. The characteristics of the parents according to HPV vaccination and intention to vaccinate their children are described in Table 2. Out of the 666 parents, only 67 (5.97%) parents were themselves vaccinated against HPV, and 91 (8.11%) had the intention to vaccinate themselves against HPV in the future. The participants with children eligible for HPV vaccination had one, two, three, or even four children. Altogether, there were 609 children, but 303 were aged 9 to 15 years. Out of these 303 children, 75 (24.75%) children were HPV vaccinated: 69 girls and 6 boys.

In the category of people with children, we carried out a multinominal logistic regression, having as a dependent variable the decision to vaccinate children, Table 3, and as independent variables, job, education, gender, age category (under 26 and over), infected with HPV (doesn’t know, no, yes), main information sources regarding vaccine (mainly medical, other), reasons of reticence (lack of information and fear of adverse effects, price, and other), and status of vaccination of the respondent. To be consistent, we took out 39 persons, who have just one child with an age below 9 years, which is the limit for the HPV vaccine, and applied regression on the remaining individuals (666 − 39 = 627). The pseudo-R square (Nagelkerke) of the model was only 0.33, which signifies that the model can explain just 33% of the attitude regarding children’s vaccination. Many other factors can be involved. The following independent variables had a statistical significance in relation to the dependent one (non-significant are not presented), Table 3.

Men have greater chances to be in the non-vaccinating category than women. Additionally, receiving information mainly from non-medical sources implies greater chances of not vaccinating than receiving information from medical sources. Having finished just high school compared to a university degree put respondents more in the non-vaccinating category than in the vaccinating. Lastly, reticence regarding vaccination because of the high cost of the vaccine and because there is a lack of info about eventual adverse effects is also a very important predictor for not vaccinating children, compared with reticence for other reasons. Interpretation for the second category (intention versus vaccination) is similar to the previous case, but with a new factor, infection of the parent, which appears to be significant. HPV-infected patients have greater chances to vaccinate their children than just having the intention to do so.

Altogether, the predictive factors for HPV vaccination of the children in the parents’ group were studies, occupation, and even the HPV infectious status of the parent. The HPV vaccine price was of great importance as well as the lack of information and the level of education of the parent.

In one group of parents, there were 67 (5.97%) parents that vaccinated their children, Table 4, and were themselves all HPV vaccinated. Most of the parents that were HPV vaccinated and vaccinated their children were women over 42 years from an urban area and working in the medical domain. In Romania, HPV vaccination is possible only with parental accord because the HPV vaccine is optional.

We tried to make a profile of the HPV-vaccinated parent that agreed to vaccinate his child: a woman from an urban area, over 42, working in the medical domain with a university degree. We found a statistical association between the fact that one individual is a parent and is HPV vaccinated, *p* < 0.001, so it is more likely that a vaccinated parent will vaccinate his/her child. Additionally, we noticed that there was a statistically significant association between the recommendation to HPV vaccinate and HPV vaccination; all the individuals that were vaccinated also recommended vaccination, *p* < 0.001.

Another category of responders that should have been more likely to vaccinate against HPV within the 1122 studied group was given by age. There were 282 responders that were of the eligible HPV vaccination age themselves, under 27 years of age. Out of them, only 2 were HPV vaccinated, and 43 intended to vaccinate against HPV. No one had children, and their main occupation was student, in the non-medical domain. Their characteristics are in Table 5.

Another aim of our study tried to expose the level of information and the factors that were conducted to this low level of HPV vaccination which are the barriers and facilitators of the current HPV vaccination Romanian campaign. As previously said, of the 1122 total responders, only 118 were vaccinated against HPV, Table 1. One of the questions that reveals a statistical significance was “Should HPV vaccination be mandatory along with the other vaccines included in Romanian National Vaccination Program (N.V.P.)?”. We found a statistically significant association between gender and the individual belief that HPV vaccination should be included in the National Vaccination Program: *p* < 0.001, 756 (75.68%) of the women vs. 84 (68.29%) of the men considered that the HPV vaccine should be mandatory. The same association was encountered regarding the recommendation of HPV vaccination, 843 (84.38%) women vs. 96 (78.05%) men. We noticed that being a woman was in favor of HPV vaccination, *p* < 0.001. In fact, more individuals of the feminine gender were vaccinated compared with the masculine individual gender, *p* < 0.001.

Some of the study variables, after stepwise selection, were significantly associated with HPV vaccination in the 118 of the group of responders that were HPV vaccinated. From the logistic regression analysis, Table 6, gender (ORa 0.461 = 95% CI: (0.196; 1.083)), geographic area of residence (ORa = 0.517; 95% CI: (0.331; 0.807)), and the inclusion of the HPV vaccine in the National Vaccination Program (ORa = 2.4; 95% CI: (1.361; 4.235)) were the factors found most associated with HPV vaccination in this group. The inclusion of HPV vaccination in the National Vaccination Program seems to be the most important factor for the level/degree of vaccination in the whole group.

## 4. Discussion

HPV vaccination is important and was a controversial subject at one time both worldwide and in Romania. The HPV vaccine is not currently mandatory or included in the National Romanian Vaccination Program [15].

Initially, the 2008 Romanian HPV vaccination campaign, when a free HPV vaccine was offered, failed, with a coverage of 2.57%, followed by the 2009 Romanian HPV vaccination campaign suspended in 2011 due to low coverage. In 2013, there was another HPV vaccination attempt in Romania, again with low vaccination coverage. All previous HPV vaccination campaigns implemented in Romania failed. It is important to ask why the coverage failed at that time. Everybody suspected that the poor acceptability was generated by poor vaccination strategies for HPV prevention [1], which means poor knowledge and acknowledgement of the HPV vaccination importance in the general population at that moment. At that time, there were enough HPV vaccine doses available, there was adequate diffusion, there was enough medical staff to administer the vaccine, and there was accessibility to the majority of the vaccinable population as the vaccination was implemented in schools. We believe that these are things that need to be remembered when the re-implementation of an HPV vaccine campaign is considered.

In trying to lower cervical cancer incidence in Romanian women, in 2016, the Romanian Ministry of Health launched The National Cancer Control Plan, and a media HPV vaccination campaign appeared for the first time in 2017 [1]. Unfortunately, the vaccine was no longer free of charge, and two or three doses, depending on the age of the vaccinee, were supposed to be bought in order to vaccinate against HPV. Since 2020, free HPV vaccination stopped for Romanian people. Another drawback was the fact that, initially, the Romanian Ministry of Health supported only the free vaccination of girls with ages between 9 and 14 years of age. Since 2020, in parallel with the COVID-19 pandemic, the Romanian Ministry of Health started again a free HPV vaccination campaign, unfortunately without a media campaign and in the background of a pandemic. Because demands increased for HPV vaccines, due to waiting lists that family doctors gathered for girls during 2016–2020, the Romanian Ministry of Health extended free HPV vaccination for girls from 14 to 18 years and started to recommend HPV vaccination in boys, but without giving the free HPV vaccine to the male gender individuals [13].

The World Health Organization has adopted a global strategy that aims to accelerate the elimination of cervical cancer for the period 2020–2030. The global strategy aims for 90% of girls to be fully vaccinated with the HPV vaccine by age 15, 70% of women to undergo a high-performance test by age 35 and again by age 45, and 90% of women identified with cervical disease to receive treatment [20].

In Romania, parents’ agreement to HPV vaccination is crucial until 18 years of age in order to reach this goal. For this reason, parents’ beliefs and HPV vaccination acceptance are critical to a successful HPV vaccination program. The present study investigates the barriers and facilitators of the present HPV vaccination campaign in Romania, where, despite HPV vaccination recommendations, HPV vaccination uptake remains low. Understanding the Romanian parents’ profile is essential for optimizing vaccination programs in Romania and improving vaccination rates.

In the present study, we noticed that the Romanian population is very confused when it comes to the importance of the HPV vaccine. Although the majority of interviewed Romanians are aware of HPV and related cancers (1023 (91.18%)) and believe that the HPV vaccine poses no risk (823 (73.35%)), only 324 (28.88%) received their HPV information from the medical doctors, and only 118 (10.51%) are HPV vaccinated. People that receive information from medical sources are the ones more likely to be HPV vaccinated or to recommend HPV vaccination as well as the younger people similar to a recent study developed at the same time as ours in Germany, where younger individuals (age group 25–34) were significantly more aware of HPV (*p* < 0.001), likely as they were offered and/or had received the HPV vaccination [20]. Even so, the number of vaccinated (adults or children) is extremely small in our country and cannot be counterbalanced by those who intend to do it and for whom there is no certainty that they will do it.

In the last couple of years and more during the COVID-19 pandemic, there was an obvious decrease in HPV vaccination rates along with a significant lack of awareness of HPV and HPV vaccination in many countries worldwide, such as: Germany [21], Austria [22], Italy [23], Saudi Arabia [24], United States [25,26], Japan [27], and Spain [28], as well as in Romania [1,12,13,16].

In Romania, women are most likely willing to be HPV vaccinated; as revealed in our study, 126 (11.22%) women vs. 6 men (0.53%), (*p* < 0.001) are HPV vaccinated, and the trend is the same in international studies; for example, in a similar study of 1035 responders in China, a total of 55.9% of males and 80.4% of females indicated that they would be willing to receive the HPV vaccine, a significant difference (*p* < 0.001) [29], but the percentages differ drastically.

The HPV vaccine is available in our country from family doctors and Public Health Directorates as the last Ministry of Health communication announced on 31 August 2022, [13]. Following the demand for the HPV vaccine, the Ministry of Health stated that in the first 6 months of this year, 2022, 52,066 doses of the HPV vaccine were used in the free vaccination campaign of the Ministry of Health. In Romania, for children up to 14 years old, two doses are required, and for those who exceed this age, three doses are used. Additionally, now, vaccination against HPV is free and is carried out in family doctors’ offices for girls between the ages of 11 and 18 based on submitted requests. The requests must come from parents and are centralized in chronological order of the registration date, and the territorial public health directorate is asked quarterly for the number of vaccine doses required for immunization. Between 1 January 2022 and 31 July 2022, a number of 30,201 vaccination requests were registered at family doctors’ offices (16,545 requests for HPV vaccination for girls aged 11–14 and 13,665 requests for HPV vaccination for girls between 15–18 years) according to the report on HPV vaccination carried out by the National Institute of Public Health—National Center for Surveillance and Control of Communicable Diseases [13].

HPV vaccination is the best method of preventing cervical cancer, and the Ministry of Health encourages parents to request information from family doctors and specialists regarding the benefits of this type of vaccination. So, since for HPV vaccination until 18 years of age parental consent is needed in Romania, an important factor that may raise the HPV vaccination rate in our country may be the parent. In Austria, for example, consent to receiving vaccines is regulated at the federal-state level, and, for example, in Tyrol, children aged 14 years are allowed to consent to receive vaccination [21]. In our study, we found out that women, female parents, are more likely to vaccinate their children against HPV *p* < 0.001.

Older studies showed that parents tended to postpone HPV vaccination as they associate HPV vaccination with sexual life or the hesitancy of medical recommendations for an early HPV vaccination [30]. One study from Poland revealed that Polish parents have a positive attitude towards vaccination; however, only 5% of daughters and 4% of sons were vaccinated against HPV in Poland [31], compared with our data, where more parents vaccinated their children. In our study out of 666 parents, 67 (10.06%) parents vaccinated their children, both girls and boys, against HPV. There are studies that associated HPV vaccination with the place of residence, more parents from urban areas would HPV vaccinate their children compared with parents from rural areas [32]; the same is true in our study.

However, the percentages of HPV vaccination are very small compared with the WHO desiderate for 2020–2030 [20]. Barriers specifically related to the HPV vaccine were recently reported in a systematic literature review [33]. For example, some parents are concerned about the effect the HPV vaccine will have on their child’s sexual behavior. As with other recommended childhood vaccines, physician recommendation for the HPV vaccine was an important component of parental support for vaccination.

The fact that most parents who said they heard about the vaccine learned about it from their doctors suggests that they acquired their medical knowledge from different healthcare experiences, underlying the lack of medical HPV vaccination publicity and media campaigns and a critical need for better public education.

Additionally, consistent with previous reviews, medical doctors’ recommendations for HPV vaccination appear to be an important clue for HPV vaccination acceptance and vaccination in general [16]. Because a medical doctor’s recommendation is critical to HPV vaccine uptake, medical doctors should receive more education on vaccine communication with parents and youths.

Perceived barriers to vaccination, including doubts about vaccine efficacy, cost, and fear of safety and side effects are usually the most cited reasons for vaccine unacceptability in the literature [30,33]. Here, in our study, we encountered the fact that if the HPV vaccine was included in the National Vaccination Program, this would raise HPV vaccination, so this may be the most important factor for the success of the present HPV vaccination campaign in our country.

Without government-funded immunization programs, cost and financial barriers were common reasons for refusing HPV vaccination and vaccination in general in the past in Romania as well as in other countries in the world [34,35,36,37,38,39,40]. Without universal healthcare coverage, the financial cost of the HPV vaccine could remain an important barrier to vaccination in some countries. The lack of a government-funded HPV vaccination program and the cost of vaccination would lead to a continued decline in HPV vaccine uptake, leaving many target populations vulnerable to possible future HPV infection [41].

Health literacy is related to an individual’s ability to obtain, process, and understand basic health information and service needs and make appropriate health decisions [42]. Parents with higher education are more likely to have better health literacy; therefore, they would have more rational attitudes toward the risks and benefits of vaccination and are more willing to be vaccinated [43,44].

Considering the problems most frequently described in recent international studies as the cause of non-vaccination [45,46,47], we note the lack of information and confusion regarding adverse effects, which can be solved through education. Recommendations for HPV vaccination coming from professional is also important [46].

However, also, the lack of a free HPV vaccine may be important, as HPV vaccination requires at least two doses and is quite expensive.

We believe that education is not enough if vaccination is not supported by the state, at least for the age groups most suitable for HPV vaccination.

Some practical implications of this study. Perhaps from the information found in our study, one example—HPV vaccination was positively influenced by gender—may be considered and be used and processed at the national level in the health policies developed by the Ministry of Health for the development of new strategies for the implementation of HPV vaccination strategies that are compliant with what the WHO specifies; i.e., a gender-neutral HPV vaccine implementation strategy. Practically, this study shows the authorities in Romania responsible for developing vaccination strategies how exactly the situation is presented in terms of gender reporting in our country and something useful in recalibrating and rethinking future vaccination strategies. The results of the study can be used in meta-analyses at the European level. The positive and negative factors that influence vaccination can be different from one country to another. Therefore, the study can be a small but representative part of the global or European situation, and based on them, along with other studies of this type, the factors that positively or negatively affect HPV vaccination can be identified, and some vaccination implementation strategies can be refined, maybe at a higher level.

### Limitations of the Study

This study was subject to several potential limitations. The study was limited by its cross-sectional design. The representativeness of the study sample is limited due to the default selection of respondents who have digital skills, as the studied sample mainly analyzed people who could use the internet and electronic documents. Another limitation of this study is that it analyzed only a short period of time of 4 months and included a limited number of participants with children (666, 59.36%). It is possible that people’s acceptability of child vaccination may change over time as further HPV vaccination campaigns may be instituted; therefore, longitudinal monitoring is indicated. Third, there are some limitations regarding the place of residence, as 930 (82.89%) of our studied group lived in an urban area, and 459 (40.91%) of the responders worked in the medical domain. Nevertheless, this may not, in fact, be a limitation because these people are opinion formers and may support children’s vaccination.

## 5. Conclusions

The findings of this study reveal the barriers and facilitators of Romanian HPV vaccinations. In the general population, the inclusion of HPV vaccination in the National Vaccination Program would be the most important facilitator of HPV vaccination in Romania. In general, HPV vaccination in Romania was positively influenced by gender and area of residence. In the case of parents who have not vaccinated their children against HPV, one important barrier to HPV vaccination was the high cost of the HPV vaccine and the lack of proper information. The overall acceptability of HPV vaccination in the Romanian population is low. Therefore, public health educational programs are needed along with the inclusion of HPV vaccination in the National Vaccination Program, thus making the HPV vaccine free of charge and available for boys as well as for girls of HPV vaccination-eligible age.

## Figures and Tables

**Table 1:** Vaccination/infectious HPV status, socio-demographic, and educational characteristics of respondents to the questionnaire. HPV awareness.

Characteristics	N = 1122 (100%)
Vaccinated against HPV	118 (10.51%)
Intention to vaccinate against HPV	132 (11.76%)
Infected with HPV	99 (8.82%)
Gender (Women)	999 (89.04%)
Age (median, min-max)	38 (19–62)
Responders < 27 years	282 (25.13%)
Geographic area (Urban)	930 (82.89%)
Domain of activity: medical	459 (40.91%)
Domain of activity: student	206 (18.36%)
Studies	
High School	249 (22.19%)
Post-secondary school	186 (16.58%)
University/Post-university studies	684 (60.96%)
Have children	666 (59.36%)
Have heard of HPV	1023 (91.18%)
Have HPV information from medic	324 (28.88%)
HPV vaccine poses no risk	823 (73.35%)

**Table 2:** Infectious HPV status, socio-demographic and educational characteristics of the 666 Romanian parents according to HPV vaccination and intention to vaccinate their children.

Characteristics	Did Not Vaccinate against HPV Their Child/Children	Intention to Vaccinate against HPV Their Child/Children	They Vaccinated against HPV Their Child/Children	*p*
Age (average ± standard deviation)	44 ± 7	39.64 ± 6.9	43.2 ± 7	0.00
Women	321	231	54	0.645
Studies				
High School	27	12	6	0.093
Post-secondary school	93	45	15	
University/Post-university studies	228	198	39	
Geographic area (Urban)	276	2016	45	
Domain of activity: medical	180	102	27	0.016
Infected with HPV	21	33	3	
Have heard of HPV	315	249	6o	
Have HPV information from medic	138	138	44	
HPV vaccine pose no risk	207	222	52	
HPV vaccination mandatory	150	30	15	0.00
High cost of HPV vaccine	57	78	6	

**Table 3:** Multinominal logistic regression in the category of people with children.

Responders who did not vaccinate versus responders who vaccinated		**ORa**	**95% CI**
	Man versus women	0.218	[0.064–0.742]
	Highschool versus university (studies)	0.107	[0.026–0.444]
	Non-medical information sources versus medical	0.035	[0.004–0.314]
	Reticence because of lack of information versus reticence from other causes	22.14	[9–54]
	Reticence because price versus reticence from other causes	7.8	[2.7–22.3]
Responders intended to vaccinate versus responders who vaccinated		**ORa**	**95% CI**
	Non-medical occupation versus medical occupation	0.156	[0.031–0.784]
	Highschool versus university	0.122	[0.028–0.533]
	Not infected versus infected	0.278	[0.074–0.99]
	Reticence because of lack of info versus reticence from other causes	12.22	[4.95–30.1]
	Reticence because of price versus reticence from other causes	14.6	[5.18–41.2]

**Table 4:** Vaccination/infectious HPV status and socio-demographic and educational characteristics of HPV-vaccinated parents that vaccinated their children against HPV.

Characteristics	N = 67
Infected with HPV	18 (26.87%)
Gender (Women)	67 (100%)
Age (median, min-max)	42 (28–55)
Geographic area (Urban)	58 (86.57%)
Studies	
High School	6 (8.96%)
Post-secondary school	10 (14.93%)
University/Post-university studies	51 (76.12%)
Domain of activity: medical	45 (67.16%)

**Table 5:** Vaccination/infectious HPV status and socio-demographic and educational characteristics of respondents with ages less than 27 years.

Characteristics	N = 282
Infected with HPV	21 (7.44%)
Gender (Women)	279 (98.94%)
Age (median, min-max)	22 (19–26)
Geographic area (Urban)	225 (79.79%)
Vaccinated against HPV	30 (10.64%)
With Intention to Vaccinate against HPV	93 (32.98%)
Studies	
High School	198 (70.21%)
Post-secondary school	6 (2.13%)
University/Post-university studies	78 (27.66%)
Domain of activity: medical	30 (10.64%)
Domain of activity: student	206 (73.05%)

**Table 6:** Logistic regression model for the 118 HPV-vaccinated responders of our study.

	B	E.S.	Wald	*p*	ORa	Lower CI 95%	Upper CI 95%
Age	−0.242	0.247	0.959	0.327	0.785	0.483	1.275
Gender (woman vs. men)	−0.774	0.436	3.154	0.076	0.461	0.196	1.083
Studies (university and more vs. high school and secondary school)	−0.213	0.220	0.945	0.331	0.808	0.525	1.242
Geographic area (urban vs. rural)	−0.660	0.228	8.416	0.004	0.517	0.331	0.807
HPV vaccination to be included in N.V.P. (yes vs. no)	−0.876	0.290	9.139	0.003	2.4	1.361	4.235
Constant	−2.085	0.345	36.572	0.000	0.124		

## Supplementary Materials

The following supporting information can be downloaded at: https://www.mdpi.com/article/10.3390/vaccines10101722/s1. File S1: Study on hpv vaccination and fear associated with this vaccination; File S2: Questionnaire Response; File S3: STROBE Statement—Checklist of items that should be included in reports of cross-sectional studies
