# Supplementary material for: Barriers and Facilitators of Romanian HPV (Human Papillomavirus) Vaccination
Source: Vaccines (Basel). 2022 Oct 15;10(10):1722. doi: 10.3390/vaccines10101722 (PMC9611461; doi:10.3390/vaccines10101722)
Supplement: Supplementary file 1 [file vaccines-10-01722-s001.zip › Supplementary Materials File S1.pdf]

STUDY ON HPV VACCINATION AND FEAR ASSOCIATED WITH THIS VACCINATION

INFORMED CONSENT/QUESTIONNAIRE ADDRESSED TO PERSONS OVER 18 YEARS OLD

We invite you to contribute to our research on vaccination and concerns about HPV infection. The questionnaire to be completed has several sections and is addressed both to the medical and social staff from the specialized units: doctors, social workers, medical assistants, pharmacists, nurses, dental technicians, midwives, paramedics, physiotherapists, as well as to the general population, especially parents with children of vaccination-eligible age, but also young people who are in the age category suitable for anti-HPV vaccination. The time required to complete this questionnaire is approximately 10 minutes. Participation is voluntary and your answers will be treated confidentially, the information you provide will only be used for research purposes. You have the freedom at any time to withdraw from the study, to interrupt or to resume the questionnaire.

The research complies with the international ethical recommendations regarding the absolute confidentiality of the data collected in the study as well as the anonymity and safety of the respondents' data. The requirements of EU Regulation 2016/79, for the protection of individuals regarding the processing of personal data and the free movement of such data, are respected. The requirements of Law no. 506/2004, regarding the processing of personal data and the protection of private life in the electronic communications sector are respected. It is important that during the study you answer the questions as honestly as possible to draw correct conclusions.

For any clarification related to this study, during or after its completion you can contact us by email at loredana.manolescu@umfcd.ro

I. GENERAL DATA

1. Occupation

- Student outside the medical field /- employee - outside the medical field

- employee in the medical field /- unemployed

2. Age:

3. Gender:

- female /- male

4. Completed studies:

-high school /- Post-secondary school /- University studies or Post university studies

5. Do you have children?

Yes /No

6. Number of children:

7. Do you have children under the age of 9?

Yes/ No

8. Chose age category:

-Children over 25 years old /-Girls 9-15 years old /-Boys 9-15 years old /

-Girls 15-25 years old / -Boys 15-25 years old

9. Did you know that the acronym HPV stands for Human Papilloma Virus and is a virus associated with cervical cancer and/or penis cancer?

-Yes / -No

10. Are you infected or have you been infected with HPV?

- yes /- no /- don't know

II. INFORMATION ABOUT HPV VACCINATION

1. Is the HPV vaccine an optional or mandatory vaccine in the national vaccination calendar?

-optional /-mandatory/-don't know

2. Do you know the benefits of HPV vaccination? Who informed you?

-Internet/ -family doctor or other medical professionals/-friends/-other media sources: facebook, tik - tok, intagram, TV, radio, etc/-family, relatives/- other sources: books, articles, newspapers, magazines, etc.

3. Have you been vaccinated against HPV?

-yes/ -no/ I don’t know/ I don't intend to

4. What do you think about HPV vaccination? Do you think you are subjecting yourself to a risk by vaccinating yourself against HPV?

Yes/no/I do not know

5. Have you vaccinated your children against HPV?

-yes/ -no

6. If you or your child have not been vaccinated against HPV, please say your reasons.

7. Do you agree (recommend) HPV vaccination?

Yes/no

8. Do you agree that the HPV vaccine should be a mandatory vaccine in the National Vaccination Program?

- Yes /- No
