# Supplementary material for: Barriers and Facilitators of Romanian HPV (Human Papillomavirus) Vaccination
Source: Vaccines (Basel). 2022 Oct 15;10(10):1722. doi: 10.3390/vaccines10101722 (PMC9611461; doi:10.3390/vaccines10101722)
Supplement: Supplementary file 1 [file vaccines-10-01722-s001.zip › Supplementary Materials File S2.pdf]

Questionnaire Response

Characteristic	N = 1122		
**Sex**	Feminine	999 (89.04%)	
	Masculine	123 (89.04%)	
**Age (median,min-max)**		38, 19-62	
**Geographic area**		Rural	174 (15.51%)
		Urban	930 (82.89%)
**Studies**	University studies/Post university studies		684 (60.96%)
	High School		249 (22.19%)
	Post-secondary school studies		186 (16.58%)
**Profession:**	Student outside the medical field		206 (18.36%)
	Employee - other occupation outside the medical field		411 (36.63%),
	Employee in the medical field		459 (40,9.%);
	Unemployed		46 (4.10%)

Question	yes	no	I don't know
Are you infected or have you been infected with HPV?	99 (8.82%)	831 (74.06%)	192 (17.11%)
Do you have children?	666 (59.36%)	456 (40.64%)	
Did you know that the acronym HPV stands for Human Papilloma Virus and is a virus associated with cervical cancer and/or penile cancer?	1023 (91.18%)	99 (8.82%)	
Number of children:			
Children over 25 years old	72 (6.42%)	1050(93.58%)	
Children under 9 years old	57 (5.08%)	1065 (94.92%)	
Girls 9-15 years old	156 (23.42%)		

Boys 9-15 years old	147 (22.07%)
Girls 15-25 years old	147 (22.07%)
Boys 15-25 years old	159 (23.87%)

II. INFORMATION ABOUT THE ANTI-HPV VACCINATION

Is the HPV vaccine an optional or mandatory vaccine in the national vaccination calendar?

optional	945 (84.22%)
mandatory	27 (2.41%)
I don't know	150 (13.37%)

What do you think about HPV vaccination? Do you think you are subjecting yourself to a risk by vaccinating yourself against HPV?

Yes	68 (6.06%)
No	823 (73.35%)
I don't know	231 (20.59%)

Have you been vaccinated against HPV?

Yes	118 (10.52%)
No	833 (74.24%)
I don't know	39 (3.48%)
I don't intend to	132 (11.76%)

Do you agree (recommend) vaccination against HPV?

Yes	939 (83.69%)
No	183 (16.31%)

You agree that the vaccine against HPV is a mandatory vaccine?

Yes	840 (74.87%)
No	282 (25.13%)
